# Simultaneous determination of inorganic and organic ions in plant parts of *Betula pendula* from two different types of ecosystems (Wielkopolski National Park and Chemical Plant in Luboń, Poland)

**DOI:** 10.1007/s11356-016-6274-4

**Published:** 2016-02-23

**Authors:** Marcin Frankowski

**Affiliations:** Department of Water and Soil Analysis, Adam Mickiewicz University in Poznań, Umultowska 89b, 61-614 Poznań, Poland

**Keywords:** Inorganic ions, Organic ions, *Betula pendula*, Soil, Wielkopolski National Park, Chemical Plant in Luboń, HPIC

## Abstract

The results of inorganic and organic anion concentrations in samples of soils and plant parts of *Betula pendula* (tap roots, lateral roots, stem, twigs, leaves), in the bioavailable fraction, are presented in this study. An ion chromatography method was applied for the first time in the simultaneous determination of inorganic and organic anions, as an effective tool for qualitative and quantitative analysis of samples with different matrix. A linear gradient elution with potassium hydroxide allowed for the separation of both inorganic and organic ions such as: F^−^, CH_3_COO^−^, HCOO^−^, Cl^−^, NO_2_^−^, Br^−^ and NO_3_^−^, SO_4_^2−^, CH_2_(COO)_2_^2−^, C_2_O_4_^2−^, PO_4_^3−^ and C_3_H_5_O(COO)_3_^3−^. The samples of soils and plant parts of *B. pendula* from the area of the Wielkopolski National Park (WNP) and the Chemical Plant in Luboń (LU; protected vs. contaminated area) were selected for the study. The obtained results indicated that such inorganic ions as: F^−^, Cl^−^, NO_3_^−^ and PO_4_^3−^ are quite easily transported from soil to leaves. In contrast, the mechanism of migration could not be clearly defined for SO_4_^2−^ because the ion was retained in roots of many of the analysed samples. Significantly higher bioavailability of inorganic ions was observed for samples collected from the area of the WNP. Phosphates were the only ions which showed no variation in their concentrations between the two sampling sites, both for soils and plant parts of *B. pendula*. None of the organic anions was detected in soil samples. The acetate, formate, malonate, oxalate and citrate ions were detected in all leaf samples. The statistical analysis allowed the author to determine the mechanism of ion migration and accumulation in leaves and, additionally, determine the variation in the occurrence of inorganic and organic ions depending on the sampling site (WNP vs. LU). The results of the statistical analysis were confirmed by the bioacumulation (BF) and translocation (TF) factors.

## Introduction

It is well known that soil is a natural reservoir of the elements essential for plant nutrition (Cataldi et al. 2003). The transfer of these elements from the soil to plants is not a simple physicochemical process and cannot be explained without regard to both physiological properties of plants and conditions in which plants grow (Baltrenaite et al. 2012). The most important nutritional elements necessary for the plants are phosphorus, potassium and nitrogen (consumed in the greatest amount). It was found that inorganic salts are important constituents of cell membranes; they are involved in many metabolic processes and can regulate the permeability of the membrane as well as interfere in the process of osmosis. Also, the organic acids play an important role in the plant metabolism and catabolism. For example, they are involved in the Krebs cycle (e.g. citrate, malate, acetate, succinate and fumarate), in respiration and photosynthetic processes (malate) and in detoxification (oxalate and citrate) (Truicǎ et al. 2013). Furthermore, some organic acids such as malic, citric and oxalic acid, can act as efficient chelators of aluminium—a very toxic metal in soils (Barbas et al. 1999; Frankowski et al. 2013). Plants are able to absorb micro- and macronutrients from the soil. The uptake of particular inorganic nutrients varies depending on plant species. Moreover, this uptake can be affected by the presence of different inorganic and organic compounds in soils (Cataldi et al. 2003; Barbas et al. 1999). Taking into account the complexity of both plant and soil processes, it is essential to qualitatively and quantitatively measure the major nutrients. The most frequently reported method for the determination of anions in plants and soils is the ion chromatography (Warren and Adams 2004; Stanišić et al. 2011). However, the simultaneous analysis of inorganic and organic ions is often not possible due to the co-elution of various co-extracted organic acids with inorganic anions and vice versa (Warren and Adams 2004; Cataldi et al. 2003). Therefore, the analysis of organic acids is usually conducted separately by using HPLC and GC methods, which additionally require pre-column derivatisation (Barbas et al. 1999; Warren and Adams 2004; Truicǎ et al. 2013; Chen et al. 1998). Separation of organic anions can also be conducted by capillary electrophoresis (CE) (Warren and Adams 2004). Truicǎ et al. (2013) determined five short-chain organic acids (succinic, citric, malic, tartaric and lactic) in tree types of medicinal plants by CE technique. Barbas et al. (1999) conducted the qualitative analysis of eight organic acids (oxalic, formic, fumaric, malic, succinic, citric, acetic and lactic) in root samples by applying the capillary zone electrophoresis technique (CZE). Warren and Adams (2004) determined major inorganic (nitrite, nitrate, chloride, sulphate, phosphate) and organic (oxalate, malate, citrate, succinate) anions in leaf extracts by CE technique. During the separation of organic acids, they observed broad peaks with some evidence of tailing. The good repeatability and occurrence of narrow peaks was only possible when the capillary was rinsed with 0.25 M HCl between runs (Warren and Adams 2004). Cataldi et al. (2003) determined nitrate, chloride, sulphate, phosphate, malate and oxalate anions in plant extract samples (leaves, roots) by the IC-CD method with isocratic elution. However, a slight overlapping of sulphate and malate signals was observed during the analysis of standard solutions and plant extract samples.

It can be summarised that the basic methods of ion chromatography in the analysis of inorganic ions allow for the determination of ligands such as: F^−^, PO_4_^3−^ and SO_4_^2−^. The simultaneous analysis of inorganic and organic ligands is more difficult. The use of isocratic elution does not separate oxalate or citrate ions. It should be highlighted that both inorganic and organic anions play an important role in aluminium complexing in the soil as well as in plant parts of *Betula pendula* (Frankowski 2015). The bioavailability of ligands may cause the reduction of aluminium toxicity in plants (Martin 1996; Frankowski et al. 2013; Karak et al. 2015). In consequence, the information about the availability of inorganic and organic anions is very important for plant growth.

The objectives of the present study were to: (1) develop the ion chromatography method with gradient elution for simultaneous determination of inorganic (F^−^, Cl^−^, NO_2_^−^, Br^−^, NO_3_^−^, SO_4_^2−^, PO_4_^3−^) and organic (CH_3_COO^−^, HCOO^−^, CH_2_(COO)_2_^2−^, C_2_O_4_^2−^, C_3_H_5_O(COO)_3_^3−^) anions in water extracts of soils and plant parts of *B. pendula*, (2) determine the variation of particular ions in soils and plant parts of *B. pendula*, (3) determine the impact of soil contamination on inorganic salt concentration in different plant parts of *B. pendula*, (4) calculate correlations of inorganic and organic anions in soils and plant parts of *B. pendula* and (5) describe a possible pathway of migration of inorganic and organic ions in the soil-root-stem-twig-leaf system.

## Materials and methods

### Samples

Soil and sapling (*B. pendula*) samples for the analysis were collected in September 2014. The samples of *B. pendula* (around 3-year-old trees) were divided into five plant parts: (1) twigs, (2) stem, (3) tap roots (without soil particles—removed manually), (4) lateral roots (root and root caps, without soil particles—removed manually) and (5) leaves.

In order to determine the spatial variability of anion concentrations, the study area was divided into two “critical” areas with different anion concentrations in the soil: Luboń Chemical Plant (LU) and Wielkopolski National Park (WNP). A detailed description of both areas was presented in the previous papers, e.g. Frankowski et al. (2013) and Frankowski (2015).

Soil samples for the analysis were collected in a depth profile of 0–20 cm. They were dried at room temperature. Hygroscopic substances dissolved in water were treated as an integral component of the samples. After drying, each soil sample was sieved through 2.0-, 1.0-, 0.5-, 0.25-, 0.1- and 0.063-mm mesh size sieves, in accordance with the Polish Standards: PN-ISO 565:2000 and PN-ISO 3310-1:2000, using a LAB-11-200/UP sieve shaker (EKO-LAB, Brzesko, Poland). The grain size fraction between 0.1 and 0.25 mm was predominant and was used to prepare soil extracts. Leaf samples were ground and the other parts of the trees were divided into 0.5-cm pieces. Subsequently, such subsamples were stored in polypropylene bags until extraction and mineralization. All the 0.5-cm pieces of particular plant parts were used for extraction. The extracts were prepared in a 1:10 proportion (*v*/*v*) with deionised water, for 1 h in a magnetic mixer. Then, the water extracts were placed in Falcon tubes and the ion chromatography method was applied.

### Development of ion chromatography method for simultaneous determination of inorganic ions and organic acids

**Fig. 1:**
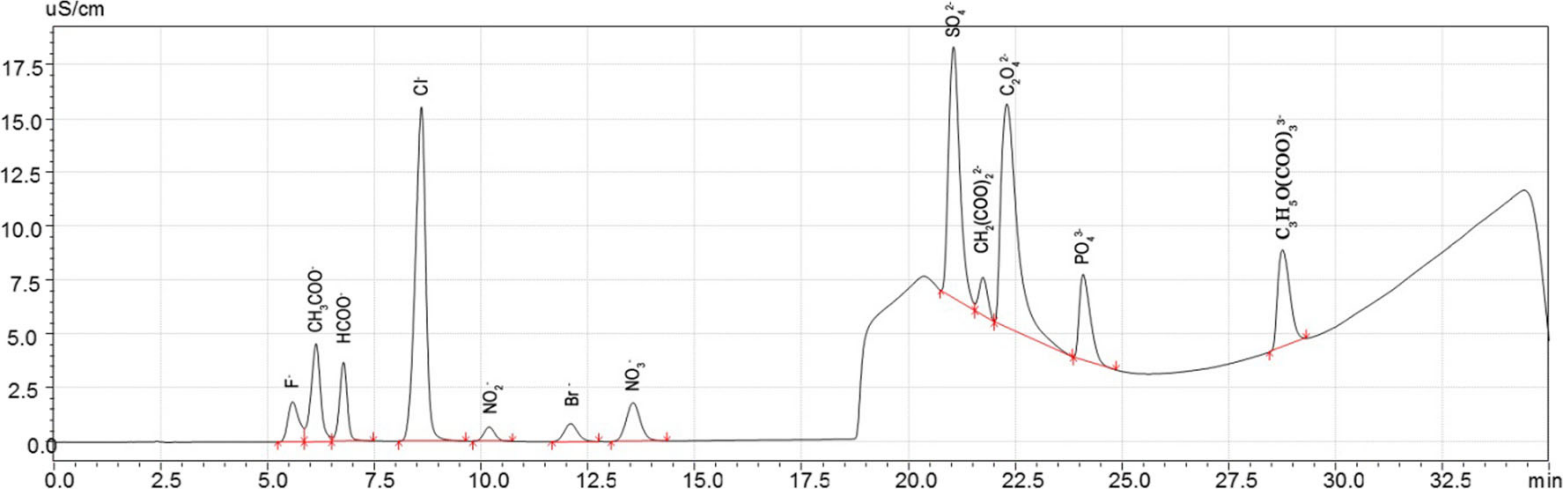
HPIC chromatogram of 12 ions with the following order of signals: (1) F^−^, (2) CH_3_COO^−^, (3) HCOO^−^, (4) Cl^−^, (5) NO_2_^−^, (6) Br^−^, (7) NO_3_^−^, (8) SO_4_^2−^, (9) CH_2_(COO)_2_^2−^, (10) C_2_O_4_^2−^, (11) PO_4_^3−^ and (12) C_3_H_5_O(COO)_3_^3−^

The analysis of inorganic and organic anions was performed using the following Shimadzu HPLC equipment: liquid chromatograph equipped with a LC-20 ADSP solvent delivery unit with low-pressure gradient flow, DGU-20A5 degasser, CDD-10Avp conductivity detector, gradient mixer (PEEK), CTO-10ASvp oven column with a Rheodyne 9725i injection valve (with a 25-μL injection loop) and Dionex A22 analytical column with a AG22 guard column. All components of this system were made from PEEK materials. The system was controlled by a CBM-20A communication bus module with LabSolution software. During the analysis, the Continuous Anion Regeneration System—CARS (SeQuant, Sweden) was used. A gradient eluent of 5–50 mM potassium hydroxide was used for determination of 12 ions. Three mixed standard solutions were prepared for this study: first mix (1.0 mg L^−1^ of F^−^, HCOO^−^, CH_3_COO^−^, NO_2_^−^, Br^−^, NO_3_^−^; 5.0 mg L^−1^ of Cl^−^, CH_2_(COO)_2_^2−^; 10.0 mg L^−1^ of SO_4_^2−^, C_2_O_4_^2−^, PO_4_^3−^, C_3_H_5_O(COO)_3_^3−^), second mix (2.5 mg L^−1^ of F^−^, NO_2_^−^, Br^−^, NO_3_^−^; 5.0 mg L^−1^ of HCOO^−^, CH_3_COO^−^; 10.0 mg L^−1^ of Cl^−^, CH_2_(COO)_2_^2−^; 25.0 mg L^−1^ of SO_4_^2−^, PO_4_^3−^; 50.0 mg L^−1^ of C_2_O_4_^2−^, C_3_H_5_O(COO)_3_^3−^) and third mix (5.0 mg L^−1^ of F^−^, NO_2_^−^, Br^−^, NO_3_^−^; 10.0 mg L^−1^ of CH_3_COO^−^; 25.0 mg L^−1^ of HCOO^−^, Cl^−^, CH_2_(COO)_2_^2−^; 50.0 mg L^−1^ of SO_4_^2−^, PO_4_^3−^; 100 mg L^−1^ of C_2_O_4_^2−^, C_3_H_5_O(COO)_3_^3−^). The separation of these ions was possible thanks to the application of gradient elution during a single analysis (Fig. 1).

As a part of method development, the following parameters: relative standard deviation (RSD), limit of detection (3 × SD), upper limit of the linear range of calibration (UL), were calculated for all 12 ions. The RSD values for all the investigated ions were below 2.5 % and the LODs were 0.03, 0.03, 0.25, 0.49, 0.19, 0.11, 0.11, 0.09, 0.06, 0.09, 0.21 and 0.21 mg L^−1^, respectively, for F^−^, CH_3_COO^−^, HCOO^−^, Cl^−^, NO_2_^−^, Br^−^, NO_3_^−^, SO_4_^2−^, CH_2_(COO)_2_^2−^, C_2_O_4_^2−^, PO_4_^3−^ and C_3_H_5_O(COO)_3_^3−^. For all the measured ions, the UL was found to be 100 mg L^−1^, except for CH_3_COO^−^ −600 mg L^−1^, HCOO^−^ −350 mg L^−1^, C_2_O_4_^2−^ −300 mg L^−1^ and C_3_H_5_O(COO)_3_^3−^ −400 mg L^−1^. The presented method was successfully applied for the determination of inorganic and organic ions in the water extracts of *B. pendula* plant parts and soils.

## Results and discussion

### Inorganic anions

#### Fluorides

**Fig. 2:**
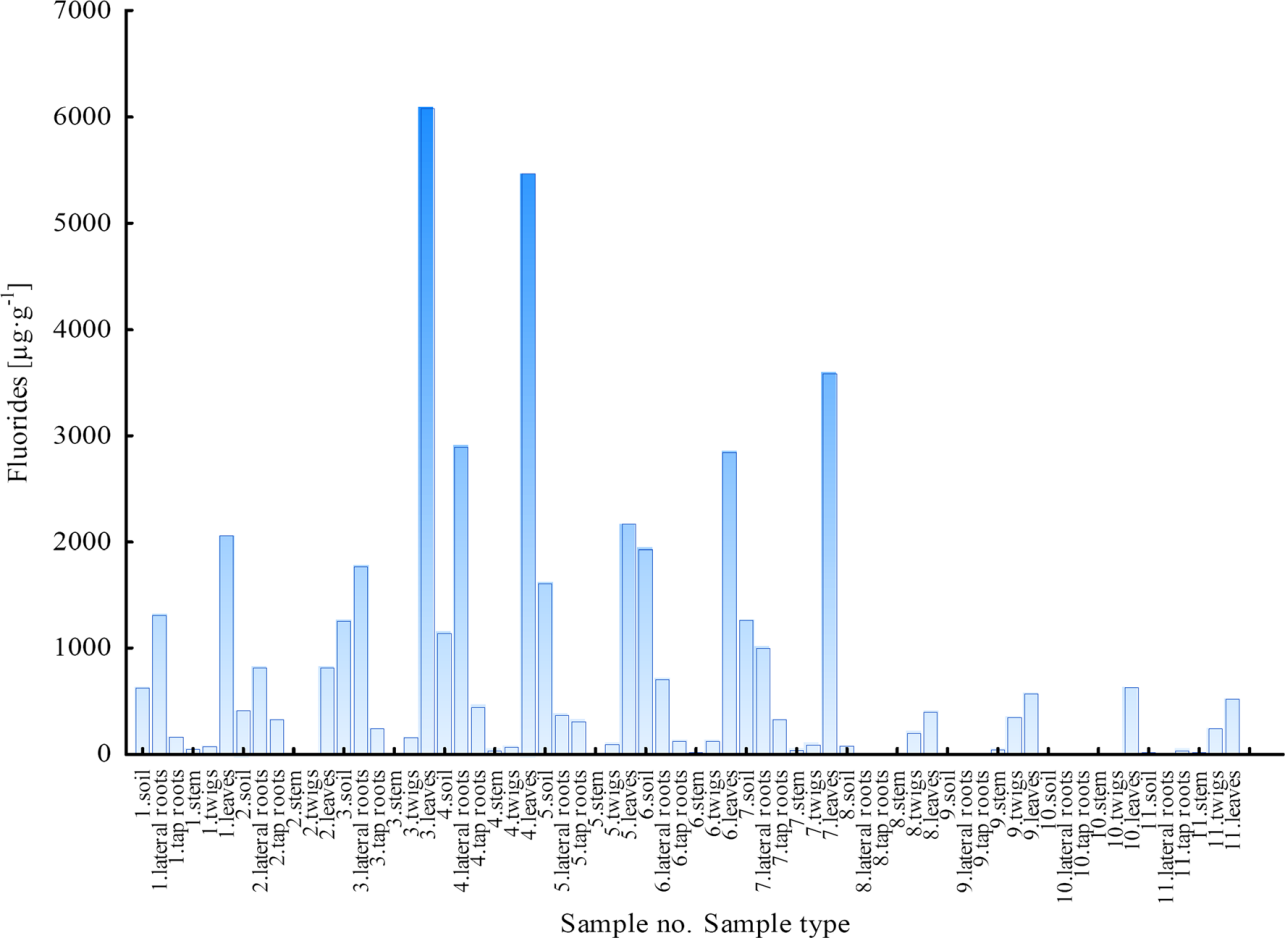
Concentration of F^−^ ions in soils and plant parts of *Betula pendula*

The results of fluoride ion concentrations for 11 samples of *B. pendula* (samples 1–7, LU; 8–11, WNP) are presented in Fig. 2. Based on the obtained results, we could clearly distinguish two groups of samples: the first one—samples collected in the LU area and the second one—samples from the WNP. For all the analysed samples of *B. pendula* plant parts, the concentration of fluorides was higher in samples taken from the LU area. Nevertheless, particular attention should be paid to the distribution of fluoride ions in different plant parts of *B. pendula*. The concentration of F^−^ was the highest in leaves from both sampling sites, which may indicate that fluorides can easily migrate from soil to leaves. This statement refers to both sampling areas; however, in the case of samples collected in the LU, the concentrations in leaves were definitely higher. The values of fluoride ions in leaves ranged from 814.3 to 6090 μg g^−1^ (average, 3290 μg g^−1^) and from 399.7 to 629.7 μg g^−1^ (average, 530.1 μg g^−1^), respectively, for the LU and WNP. The migration of F^−^ ions from soil to leaves was more evident when the concentration of these ions in soil was higher (LU site). For the samples taken from the WNP area, it was also observed that fluoride ions could accumulate with time, which was confirmed by F^−^ concentrations determined in leaves and lower F^−^ concentrations in soils and roots (lateral and tap roots). Furthermore, it was found that the highest concentrations of F^−^ in leaves corresponded to the highest concentrations in soils, lateral roots and tap roots, e.g. samples no. 1, 3, 4 and 7.

#### Chlorides

**Fig. 3:**
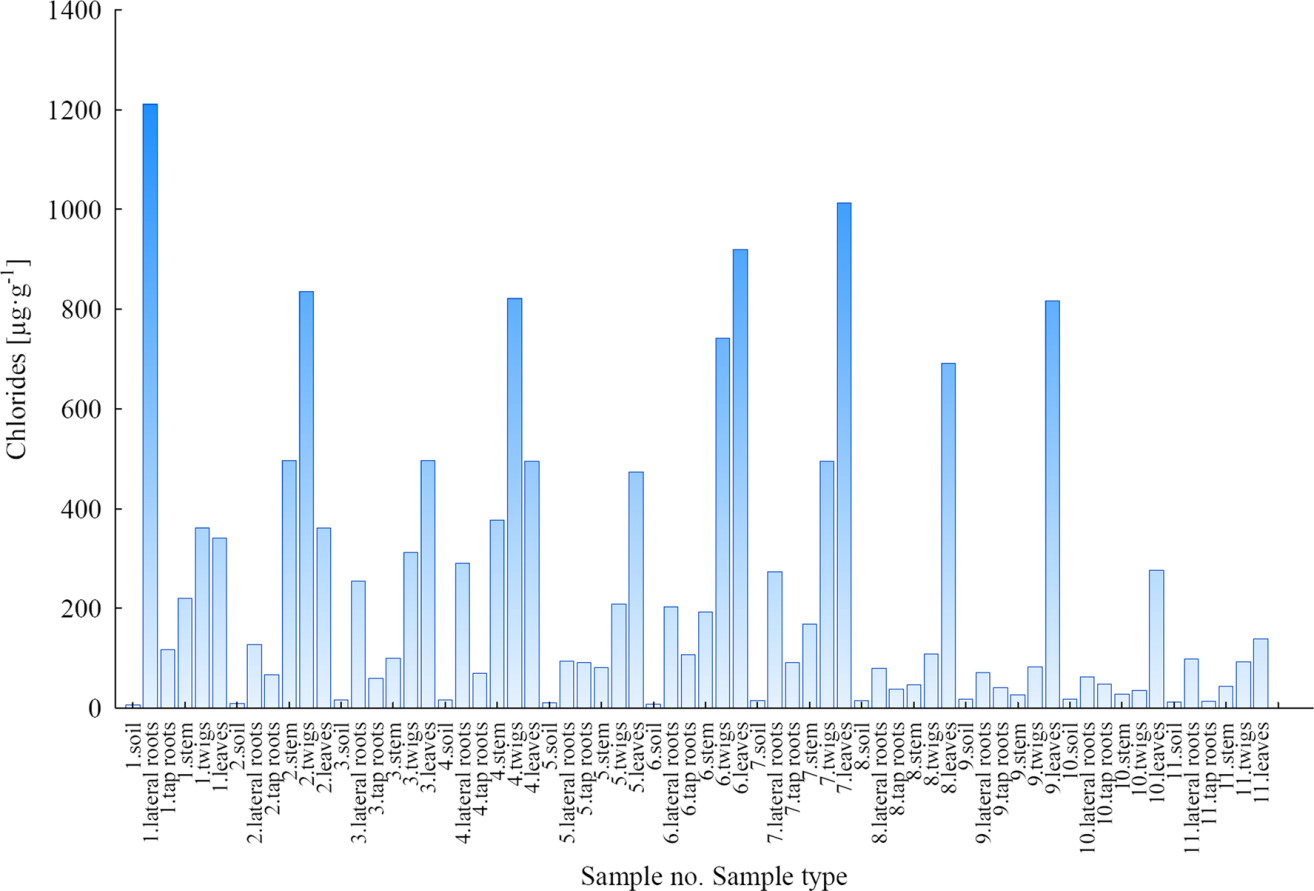
Concentration of Cl^−^ ions in soils and plant parts of *Betula pendula*

The concentrations of chloride ions in the samples of soil and plant parts of *B. pendula* are presented in Fig. 3. The lowest Cl^−^ concentrations were determined in soils collected under *B. pendula* roots. The values of Cl^−^ in soils were between 5.449 and 16.57 μg g^−1^ (average, 11.31 μg g^−1^) for the LU and between 12.37 and 17.77 μg g^−1^ (average, 15.49 μg g^−1^) for the WNP. In the collected plant parts of *B. pendula*, the concentrations were generally higher for the LU area than for the WNP area, which was the effect of relatively easy migration mechanism of Cl^−^ ions in the salt of the cation which is essential for plant growth. This was confirmed by Cl^−^ concentrations determined in leaves. They ranged from 341.7 to 1013 μg g^−1^ (average, 586.2 μg g^−1^) for samples taken from the LU and from 138.8 to 817.2 μg g^−1^ (average, 481.0 μg g^−1^) for samples taken from the WNP. It should be emphasised that Cl^−^ concentrations in the other plant parts of *B. pendula* (roots, stem and twigs) taken from the contaminated LU area were also higher than the concentrations in the corresponding plant parts from the WNP (Fig. 3).

#### Sulphates

**Fig. 4:**
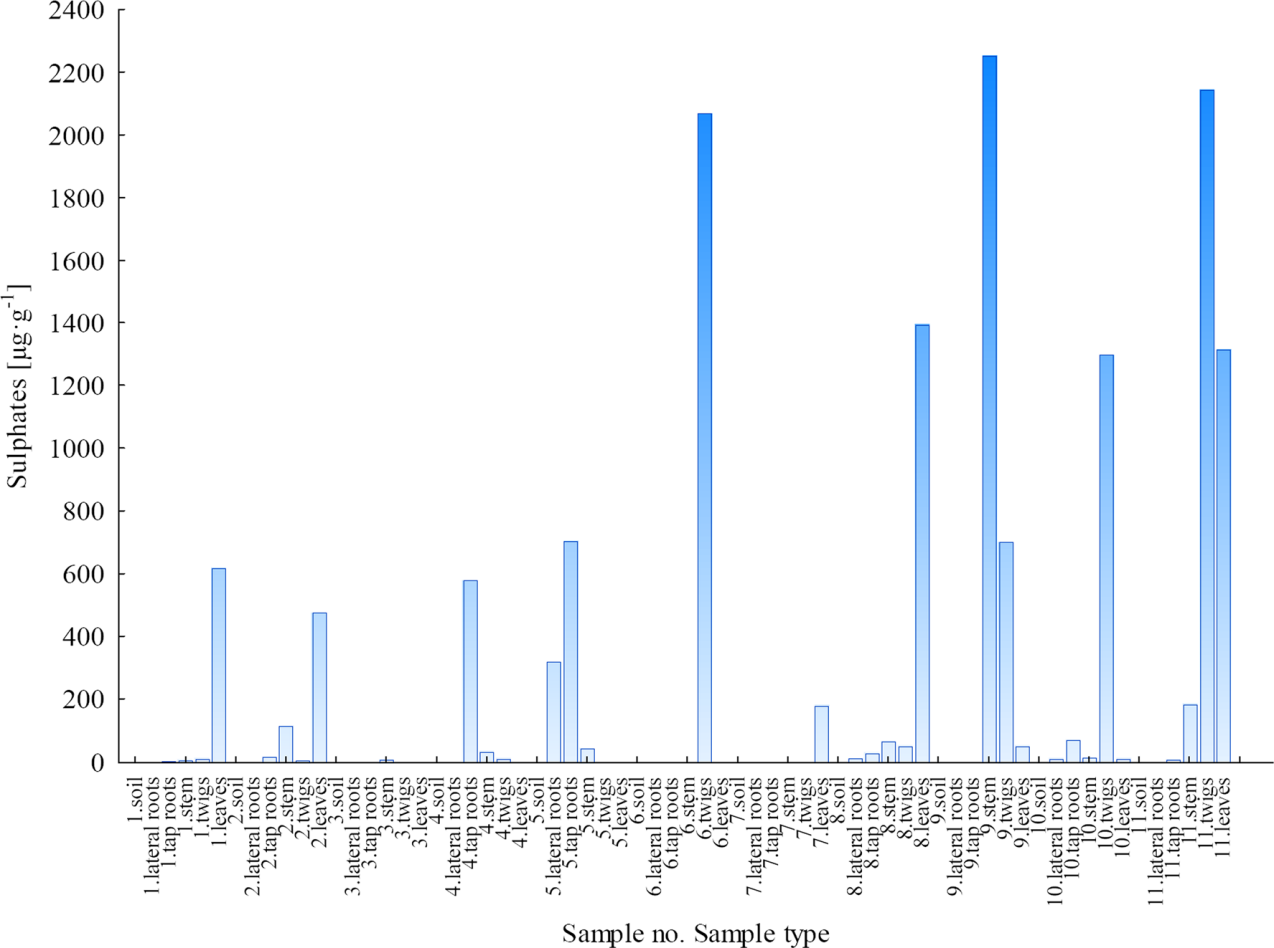
Concentration of SO_4_^2−^ ions in soils and plant parts of *Betula pendula*

Figure 4 presents sulphate concentrations in soils and plant parts of *B. pendula*. These ions were detected in 26 out of 35 samples of *B. pendula* plant parts and in all soil samples collected from the LU area. Sulphates were not detected in the following plant part samples: stem (samples 3–7), twigs (sample 5), lateral roots (sample 3) and tap roots (samples 3 and 7). For samples no. 1 and 2, the concentration of SO_4_^2−^ was determined in all the plant parts of *B. pendula* and in soils. Sulphate concentrations were below the limit of detection for soil samples collected from the WNP. They were detected only in 8 out of 20 plant part samples from the WNP. The SO_4_^2−^ concentrations were measured in samples no. 8, 9 and 11. For lateral and tap roots of sample no. 8, the values were 814.4 and 2425 μg g^−1^, respectively, while for lateral roots of sample no. 9, sulphates amounted to 130.5 μg g^−1^. The most variable concentration of SO_4_^2−^ was measured in sample no. 11: 497.6 μg g^−1^ in lateral roots, 218.5 μg g^−1^ in tap roots and 314.3 μg g^−1^ in twigs. Taking into account all the SO_4_^2−^ concentrations measured in samples collected from the LU and WNP area, it was not possible to determine the pathways of sulphate migration in the *B. pendula*. Hence, we can only speculate on the migration and transport of sulphates from soil to leaves. The majority of these ions is probably retained in lateral and tap roots. This may indicate that *B. pendula* does not need SO_4_^2−^ ions for its growth.

#### Phosphates

**Fig. 5:**
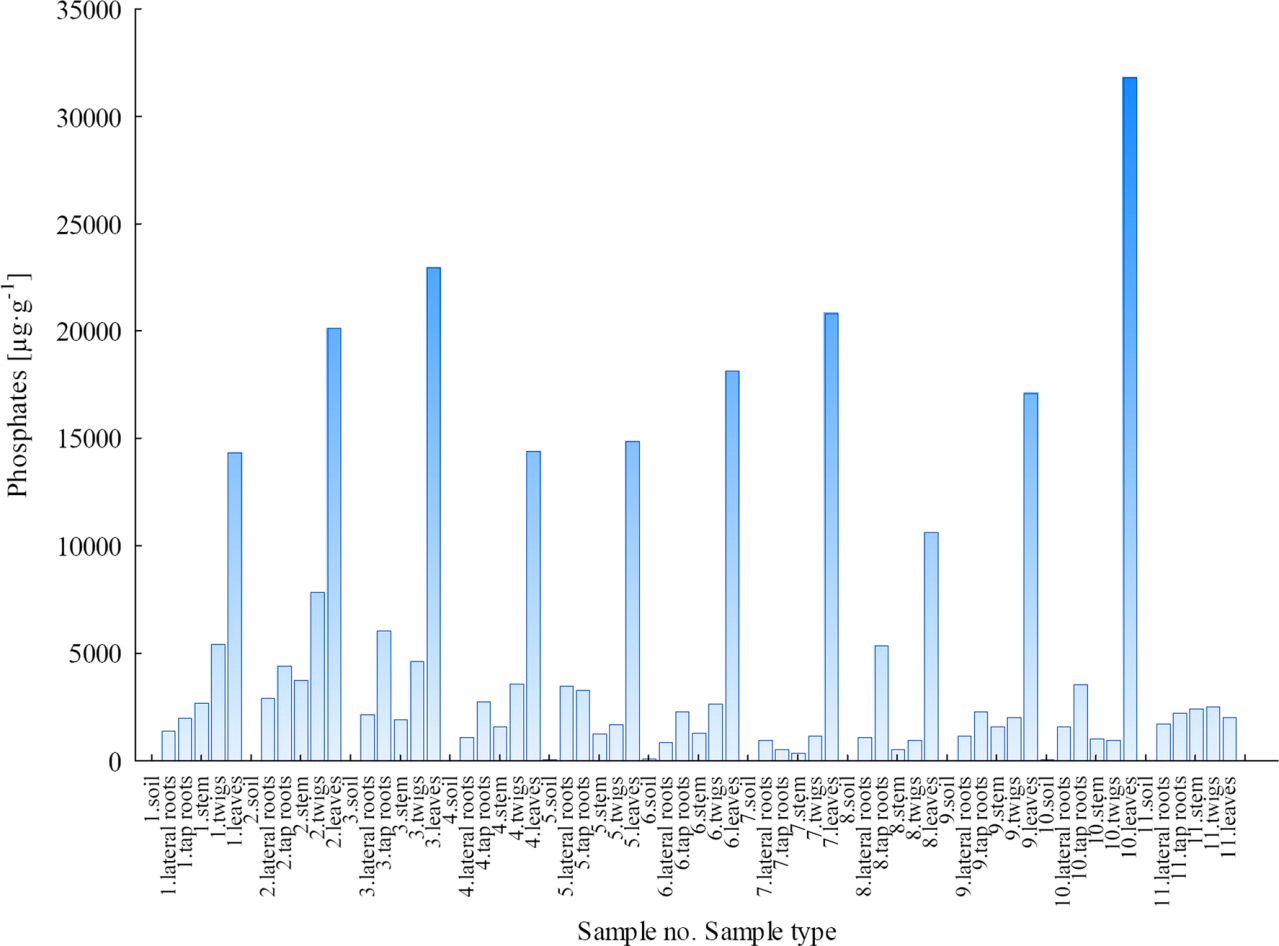
Concentration of PO_4_^3−^ ions in soils and plant parts of *Betula pendula*

In the case of phosphate ions, a completely different concentration trend in relation to F^−^, Cl^−^ and SO_4_^2−^ was observed. The results of PO_4_^3−^ concentration in all the analysed samples of soils and plant parts of *B. pendula* are presented in Fig. 5. Based on the results shown in Fig. 5, it can be concluded that the concentrations, availability, migration and transport of PO_4_^3−^ were comparable for both sampling areas (LU and WNP). Furthermore, phosphate ions were similarly distributed in the plant parts of *B. pendula* from WNP and LU, and the uptake of these ions occurred directly in the soil-leaf pathway. The highest concentrations of PO_4_^3−^ were determined in leaves, and they ranged from 14.29 to 22.94 mg g^−1^ (average, 17.91 mg g^−1^) for the LU area and from 19.94 to 31.77 mg g^−1^ (average, 15.36 mg g^−1^) for the WNP area (Fig. 5).

The variability of PO_4_^3−^ concentrations in different plant parts of *B. pendula* showed that these ions are very important for the plant, and its uptake from the soil takes place continuously. This was confirmed by the fact that concentrations of PO_4_^3−^ for most samples increased in the direction from the soil and roots, through stems and twigs, to the leaves.

#### Nitrates: NO_2_^−^ and NO_3_^−^ and Br^−^

The applied new method of ion chromatography also enabled the determination of NO_3_^−^ ions in the samples of soil and different plant parts of *B. pendula*. Based on the chromatograms, NO_2_^−^ and Br^−^ ions were not found in the samples. The separation of NO_3_^−^ form was obtained for 28 out of 66 analysed samples of soils and *B. pendula* plant parts. Nitrates were analysed in all soil samples collected from the WNP area (range, 17.48–60.66 μg g^−1^; average, 43.29 μg g^−1^) and in two soil samples from the LU area: no. 1—10.28 μg g^−1^ and no. 2—16.61 μg g^−1^. In the case of *B. pendula* plant parts, NO_3_^−^ concentrations were determined in leaves from the LU (samples no. 1–4), in five root samples (lateral and tap roots) from the LU and in two root samples from the WNP. For all the samples in which the NO_3_^−^ ion was analysed, the values were higher in tap roots than in lateral roots. The NO_3_^−^ was not measured in stem and twigs of *B. pendula*. The results of nitrate concentrations in soils and plant parts of *B. pendula* are shown in Table 1.

**Table 1:** Concentration of NO_3_^−^ ions in soils and plant parts of *Betula pendula* from LU and WNP (μg g^−1^)

Sample	Soils	Lateral roots	Tap roots	Stem	Twigs	Leaves
1	10.28	28.81	36.35	<0.11	<0.11	410.1
2	16.61	<0.11	<0.11	<0.11	<0.11	121.5
3	<0.11	<0.11	<0.11	<0.11	<0.11	9.529
4	<0.11	32.59	22.97	<0.11	<0.11	18.54
5	<0.11	24.49	3.426	<0.11	<0.11	<0.11
6	<0.11	43.76	25.72	<0.11	<0.11	<0.11
7	<0.11	123.8	16.96	<0.11	<0.11	<0.11
8	37.29	<0.11	<0.11	<0.11	<0.11	15.12
9	17.48	<0.11	<0.11	<0.11	<0.11	136.2
10	60.66	20.75	60.08	<0.11	<0.11	264.8
11	57.74	2.809	57.13	<0.11	<0.11	25.88

### Organic ions

#### Acetate

**Fig. 6:**
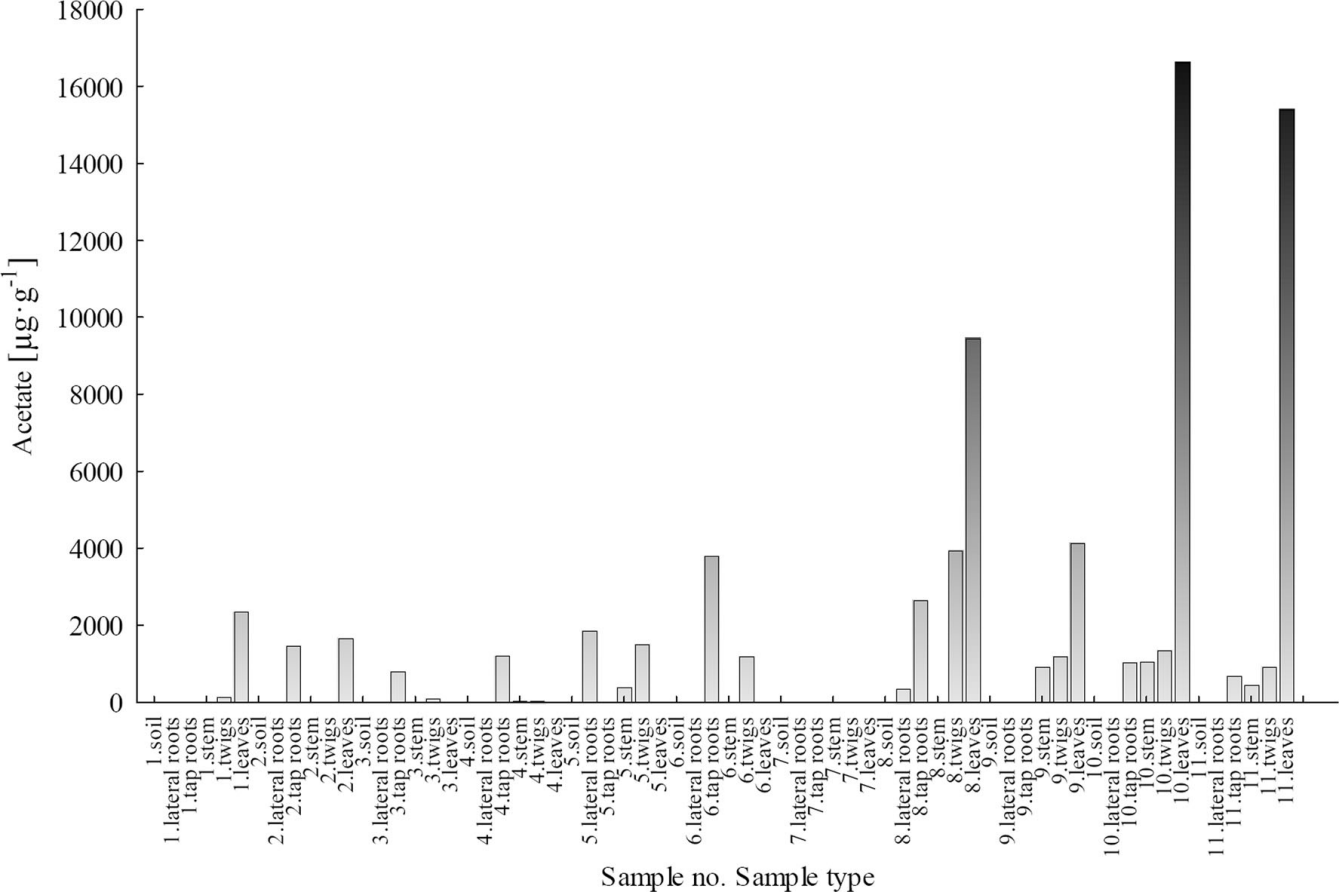
Concentration of acetate ions in soils and plant parts of *Betula pendula*

Figure 6 presents the concentrations of acetate ions in soil samples and *B. pendula* plant parts. Acetate ions were not detected in soil samples from both sampling areas: LU and WNP. The highest concentrations of acetate ions were reported for plant parts collected in the WNP area, particularly for leaf samples (4135–16,620 μg g^−1^). In the LU area, acetate concentrations were measured only in leaf samples no. 1 and 2, and they amounted to 2347 and 1673 μg g^−1^, respectively.

Acetate concentrations in the plant parts of *B. pendula* were much more variable for samples taken from the WNP than for those from the LU. Higher concentrations of acetates as well as their greater variability in the plant parts from the WNP pointed to the balance between organic and inorganic compounds available in the soil. In the case of LU samples, the balance was shifted towards inorganic forms, mainly due to contamination with inorganic compounds in the form of inorganic acids.

#### Formate

**Fig. 7:**
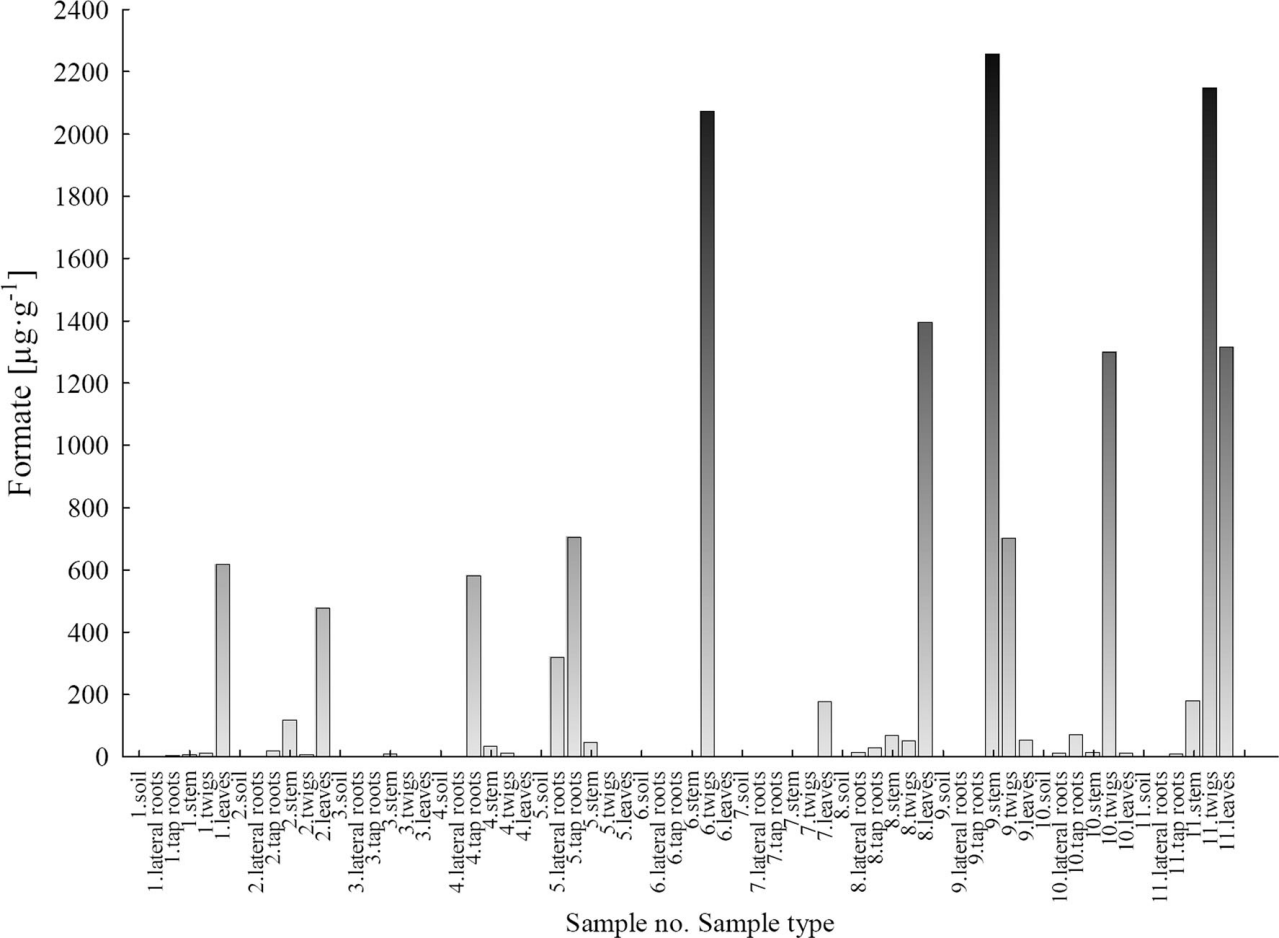
Concentration of formate ions in soils and plant parts of *Betula pendula*

Formate ion concentrations showed a similar variation trend as the concentrations of acetate ions. They were detected in the same plant part samples as acetate ions and not detected in soil samples (LU and WNP) (Fig. 7). In the LU area, formate ions were present in leaves of samples no. 1 and 2, respectively: 617.5 and 475.8 μg g^−1^. For the other plant parts of *B. pendula*, the concentration values were quite variable, e.g. concentrations in tap roots, stem and twigs of sample no. 1 were 3.3, 5.8 and 10.6 μg g^−1^, respectively, and in the same plant parts of sample no. 2, they amounted to 18.0, 116.2 and 5.1 μg g^−1^, respectively. For the other samples of *B. pendula* plant parts collected from the LU area, the following concentrations of formates were determined: sample no. 3: twigs—9.6 μg g^−1^; sample no. 4: tap roots—579.3 μg g^−1^, stem—33.0 μg g^−1^ and twigs—9.9 μg g^−1^; sample no. 5: lateral roots—318.6 μg g^−1^, tap roots—7038 μg g^−1^ and stem—45.4 μg g^−1^; sample no. 6: twigs—2070 μg g^−1^; and sample no. 7: leaves—177.4 μg g^−1^.

In the case of *B. pendula* plant part samples from the WNP, the concentrations of formates were much more variable, particularly in the stem-twig-leaf system, where the values were the highest and ranged from 14.28 to 2252 μg g^−1^ for stem, from 50.83 to 2144 μg g^−1^ for twigs and from 10.0 to 1393 μg g^−1^ for leaves. The results of formates may indicate that the soil conditions in the WNP area are better for organic matter formation than the conditions in the contaminated LU area.

The production of formate and acetate ions in *B. pendula* as well as their transport to leaves through different plant parts can be limited by inorganic ions.

#### Malonate

Malonate concentration was determined in 5 out of 66 samples of soils and plant parts of *B. pendula*. For all the five samples, malonates were detected in leaves of the plants from the LU (sample no. 1, 305.8 μg g^−1^; sample no. 3, 8.5 μg g^−1^; sample no. 4, 959.1 μg g^−1^; and sample no. 7, 343.1 μg g^−1^) and from the WNP (sample no. 8, 2444 μg g^−1^).

#### Oxalate

Oxalate ions, similarly as the organic ions discussed above, were not detected in soil samples collected under *B. pendula* plants. In plant part samples, the oxalate ions were found in all leaf samples obtained from the LU and WNP areas. The values in leaves ranged from 32.2 to 1239 μg g^−1^ (average, 477.9 μg g^−1^) for the LU area and from 45.1 to 315.9 μg g^−1^ (average, 171.9 μg g^−1^) for the WNP area. The concentration of oxalates was above the limit of detection for five additional plant part samples of *B. pendula* taken from the LU area. For sample no. 1, oxalate concentrations were as follows: 135.8 μg g^−1^ in lateral roots, 85.9 μg g^−1^ in tap roots, 108.5 μg g^−1^ in stem and 148.8 μg g^−1^ in twigs. For the other samples (no. 2, 3, 5 and 6), the values of oxalates in different plant parts of *B. pendula* were varied. In sample no. 2, oxalate ions were determined in stem and twigs (231.8 and 282.8 μg g^−1^, respectively), in sample no. 3—in lateral roots, tap roots and twigs (84.6, 14.1 and 16.68 μg g^−1^, respectively), in sample no. 5—in tap roots and lateral roots (693.1 and 169.6 μg g^−1^, respectively) and in sample no. 6—in stem and twigs (9.8 and 68.3 μg g^−1^, respectively). For samples taken from the WNP, oxalate ions were found only in two samples. In sample no. 8, the concentrations were measured in tap roots and stem (8.7 and 587.5 μg g^−1^, respectively), while in sample no. 9, the oxalates were detected only in twigs (629.8 μg g^−1^). Based on the results of oxalates in all the analysed samples, it was observed that these ions can be found mainly in leaves, and it was difficult to indicate the processes which caused that the oxalates were not determined in many samples of *B. pendula* plant parts.

#### Citrate

Citrate ions were below the limit of detection in the samples of soil, similarly as other previously described organic anions. The variation of citrate ions in the plant parts of *B. pendula* was similar to that observed for oxalate ions. Taking into account that citrate ions are larger as compared with inorganic ions, they were detected in all leaf samples collected from the LU and WNP area. The values of citrates in leaves were varied and ranged from 39.8 to 4826 μg g^−1^ (average, 973.3 μg g^−1^) for the LU and from 41.66 to 3271 μg g^−1^ (average, 872.7 μg g^−1^) for the WNP. The concentrations of citrates in the samples of leaves taken from the WNP area were as follows: 3271, 126.3, 41.66 and 51.98 μg g^−1^, respectively, for samples no. 8, 9, 10 and 11. In the case of samples from the WNP area, citrate ions were measured only in leaves, and for the samples taken from the LU area, citrates were found also in different plant parts of samples 1–5. In sample no. 1, citrates occurred in lateral roots (9.7 μg g^−1^), tap roots (3.8 μg g^−1^), stem (3.8 μg g^−1^) and leaves (79.7 μg g^−1^) in sample no. 2—in stem (64.8 μg g^−1^), in sample no. 3—in tap roots (18.6 μg g^−1^) and in stem (21.1 μg g^−1^), in sample no. 4—in tap roots (78.1 μg g^−1^) and in sample no. 5—in lateral (25.9 μg g^−1^) and tap roots (45.5 μg g^−1^). In samples no. 6 and 7, citrates were not detected in lateral and tap roots nor in stem and twigs.

#### Statistical analysis

**Fig. 8:**
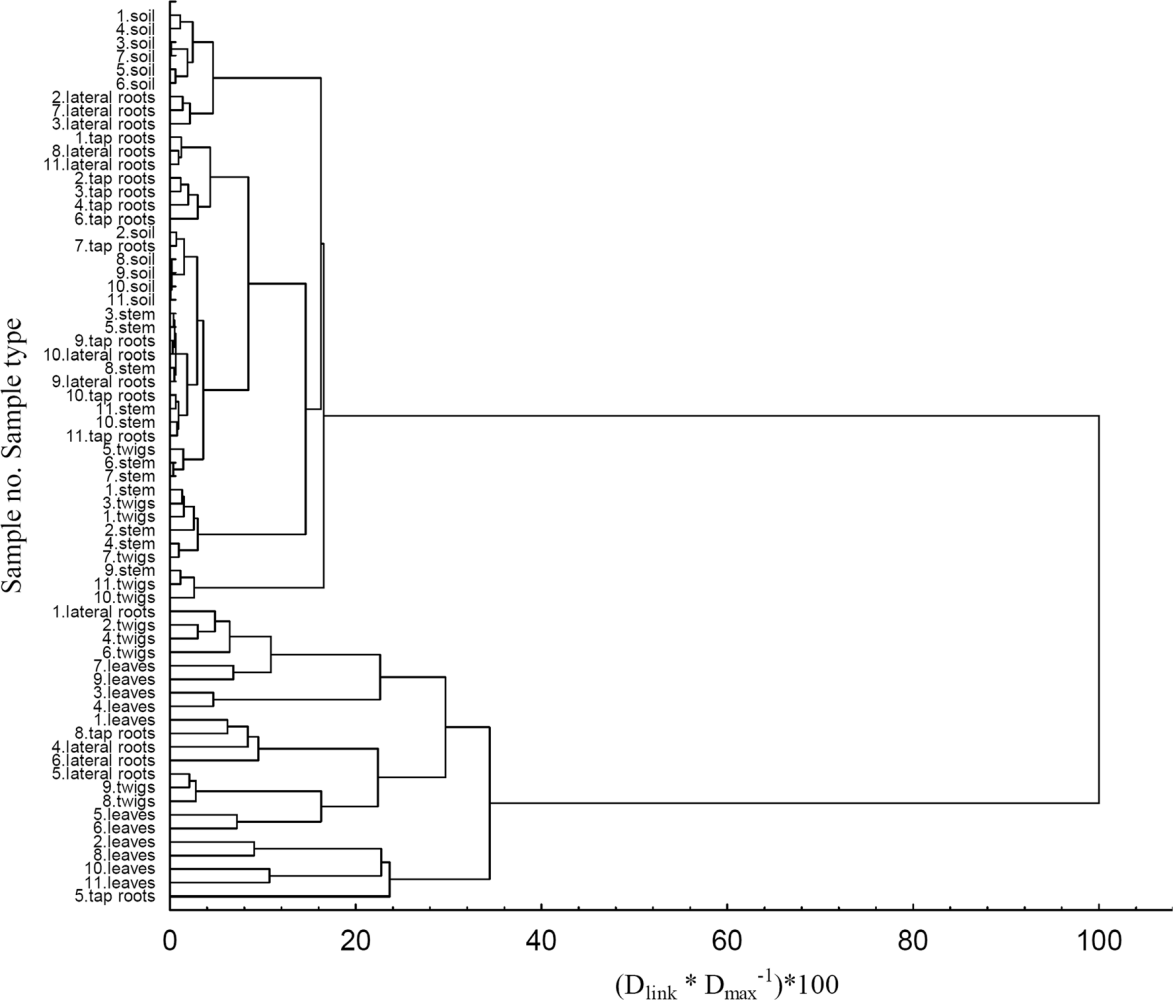
Hierarchical cluster analysis for soils and plant parts of *Betula pendula*

The ion concentrations which were below the limit of detection were complemented with values equal to half the detection limit. Then, the Shapiro-Wilk test was performed and variables with non-normal distribution were subjected to a transformation. The data set comprising of eight ions was standardised and finally analysed using three multivariate statistical methods: factor analysis (FA), cluster analysis (CA) and principal component analysis (PCA). The statistical calculations were performed using Statistica 12 software (StatSoft, Poland). The HCA method was used to determine the variability between sampling areas (LU and WNP) and between particular plant parts of *B. pendula*. Based on the HCA analysis (Fig. 8), two groups of samples were separated, which differed in the content of the following ions: F^−^, Cl^−^, SO_4_^2−^, PO_4_^3−^, CH_3_COO^−^, HCOO^−^, C_2_O_4_^2−^ and C_3_H_5_O(COO)_3_^3−^.

The groups comprised of different types of samples (soil or plant parts of *B. pendula*), which reflected the migration of ions from soil to leaves. In the first group, the samples of soil, tap roots, stem and lateral roots (7 out of 11 samples), twigs (6 out of 11 samples) and tap roots (9 out of 11 samples), from both sampling areas (LU and WNP) were clustered. The second cluster included leaf samples, also from both sampling areas and other plant part samples, i.e. lateral roots, twigs and tap roots. It should be emphasised that the groups did not differ depending on the sampling area (contaminated LU area vs. protected WNP area). The main trend of variation for the ions was related to their transport patterns, from soil to leaves, through lateral and tap roots, stem and twigs. Similar relationships in the uptake of substances from soil to leaves were reported in the study on aluminium (e.g. Frankowski 2015).

**Fig. 9:**
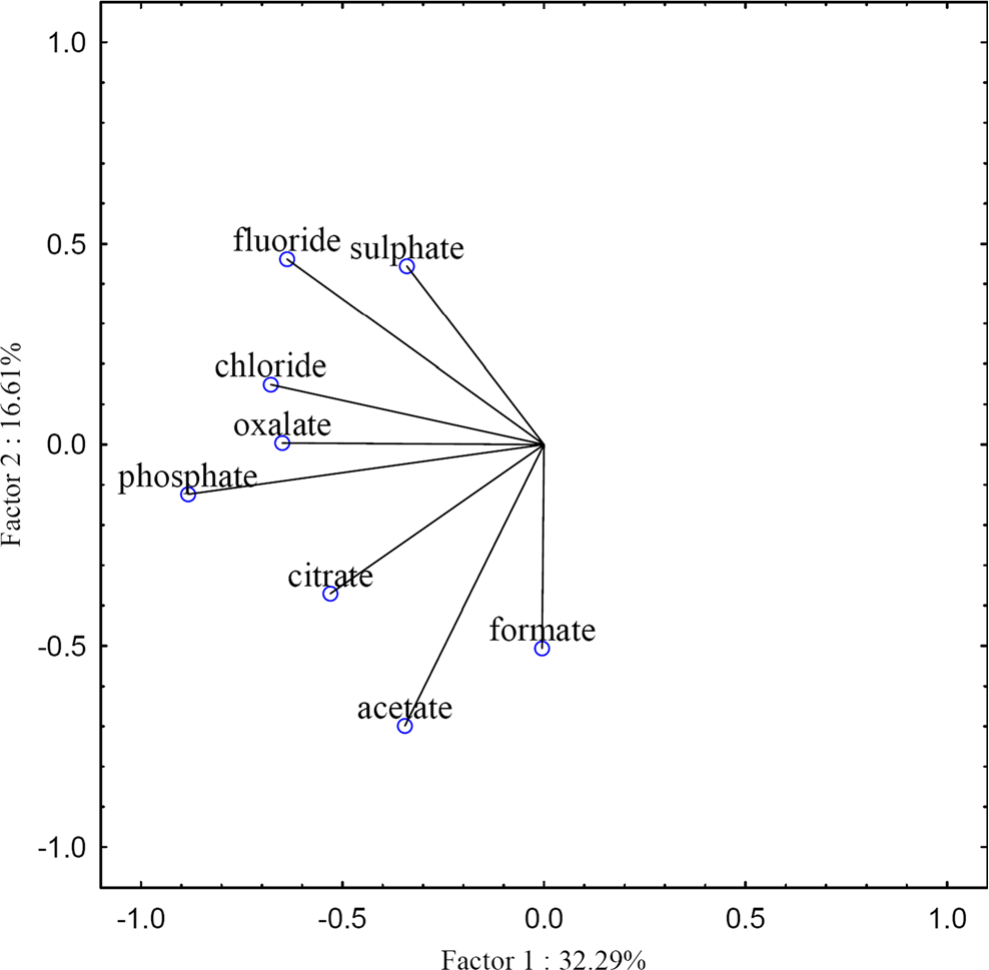
Principal component analysis of inorganic and organic anions for soils and plant parts of *Betula pendula*

In the case of the PCA analysis of the anions (Fig. 9), two different groups were separated. They comprised of inorganic and organic ions. Only for oxalate and phosphate ions, a slightly different relationship was observed. In the case of PO_4_^3−^ ions, there was no variation in their concentrations caused by the sampling area (WNP/LU). On the other hand, for oxalate ions (and partially citrate ions), the variability in the concentration of these ions in samples collected from the LU area was higher as compared with the samples obtained from the WNP area.

In the case of factor analysis, the explained variance for the analysed anions was 61.57 %, for three factors. The results of FA are presented in Table 2.

**Table 2:** Factor analysis for inorganic and organic anions in soils and plant parts of *Betula pendula*

Anion	VF1	VF2	VF3
F^−^	0.740297*	−0.016791	0.291260
CH_3_COO^−^	−0.079360	0.772277*	−0.083911
HCOO^−^	−0.548611	0.403691	0.386196
Cl^−^	0.659975	0.266080	0.143774
SO_4_^2−^	0.133719	−0.191008	0.832688*
C_2_O_4_^2−^	0.290286	0.355158	0.572830
PO_4_^3−^	0.654732	0.606248	0.181533
C_3_H_5_O(COO)_3_^3−^	0.234044	0.608043	0.049012

*Strongly correlated factor loadings

The first factor—VF1 (accounting for as much as 32.29 % of the total variance) was strongly correlated with the concentration of fluorides, which can imply a source of fluoride ions (soil and air) and the availability of these ions for both sampling areas. Higher concentrations of fluoride ions were found in air samples from the WNP area (e.g. Walna et al. 2007, 2013). The next two factors, with the cumulative variance of 29.28 %, were strongly correlated with the concentration of acetate and sulphate ions (VF2 and VF3, respectively), which indicated significant differences in relation to the other ions. This might be caused by the fact that concentrations of acetate ions in the WNP samples were higher than in the LU samples, and in the case of sulphate ions, the concentrations in the LU samples were higher (probably due to waste from the production of sulfuric acid) than in the WNP samples.

#### Bioaccumulation and translocation factors (BF and TF)

In order to compare the uptake of ions from the soil of different sampling points and the transport of ions from roots to plant parts of *B. pendula*, two additional parameters were calculated. Bioaccumulation and translocation factors (BF and TC) are important and simple parameters, which allow to estimate the uptake of metals and their translocation (Polechońska and Samecka-Cymerman 2015; Korzeniowska and Stanislawska-Glubiak 2015; Sun and Zhou 2016; Mehes-Smith and Nkongolo 2015; Mohanty et al. 2015; Pachura et al. 2015). The application of these factors for the ions analysed in the present study involved certain limitations because the organic ions were not determined in soil samples. Therefore, only the inorganic ions, i.e. F^−^, Cl^−^, PO_4_^3−^ and SO_4_^2−^, were taken into account in the calculation of BFs and TFs. The values of BFs confirmed the conclusions drawn based on concentrations of ions obtained for soils and the plant parts of *B. pendula* as well as the results of the statistical analysis. For the chlorides and phosphates, the calculated factors were >1 for all the plant parts of *B. pendula*, which suggested an easy uptake and transport of these ions. In the case of the fluorides, the calculated BFs were >1 for the following plant parts and samples: from the LU—lateral roots and leaves (all samples), from the WNP—leaves (sample 8), twigs and leaves (sample 9), stem, twigs and leaves (sample 10) and tap roots, stem, twigs and leaves (sample 11). For the SO_4_^2−^ ions, the bioaccumulation factors were >1 for all the lateral root, tap root and leaf samples and <1 for all the stem and twig samples of *B. pendula* collected from the LU area, while for the samples obtained from the WNP area, the trend in BF values was different. The translocation factor was calculated for two separate plant parts of *B. pendula*: lateral roots (concentration in plant part / concentration in lateral roots) and tap roots (concentration in plant part/concentration in tap roots). The TF values for both types of roots of *B. pendula* were significantly lower than the values of BFs. The TF values were generally higher for tap roots, which was connected with the results of BFs and anion concentrations obtained for tap roots and lateral roots. Nevertheless, despite lower TFs as compared with BFs obtained for particular anions, a similar trend of variation was observed. For Cl^−^ and PO_4_^3−^ ions, the values of TF were >1 for leaves and <1 for stem and twigs. The situation was similar for fluorides and sulphates (dependence on the area of sampling). However, in the case of SO_4_^2−^, the TF value was <1 for many leaf samples. To sum up, both BF and TF factors are good indicators for the assessment of ion bioaccumulation and translocation. However, the analysis of concentrations in particular plant parts of *B. pendula* is a more reliable analytical procedure and reflects the actual bioavailability and transport of ions and metals, from soil to leaves and from roots to leaves through particular plant parts.

## Conclusions

The application of a new ion chromatography method enabled a quick, efficient and simultaneous determination of inorganic and organic anions in the water-soluble (bioavailable) fraction of the samples of soils and plant parts of *B. pendula* from two different environments (WNP and LU). The results of the study indicated a need for further research on the mechanisms of migration and transport of ions in the soil-root-stem-twig-leaf system. Based on the study, it can be concluded that inorganic ions, i.e. fluorides, chlorides, nitrates and phosphates, are taken directly from the soil and can easily migrate to the leaves, while the migration of sulphates is more difficult. Such dependencies were found particularly for samples collected in the area of LU, where higher concentrations of inorganic ions were determined. The organic anions such as acetates, formates, malonates, oxalates and citrates were not detected in soil samples collected under *B. pendula* from the LU, which pointed to the complex mechanism of organic ion formation in *B. pendula* (they are not taken from the soil). However, organic ions were detected in all leaf samples of *B. pendula* and in some plant part samples of *B. pendula*. Based on the research, it was difficult to clearly explain the variability of organic anions in tap roots, lateral roots, stem and twigs. Higher concentrations of the analysed inorganic anions were measured in the LU samples, while for the organic anions, in particular for acetates and formates, higher values were determined in the samples taken from the WNP area. For oxalates and citrates, the variability was definitely higher for the LU area. Taking into account the fact that the analysed organic ions are not present in the soil, their concentration values obtained in this study raised numerous questions related to the migration and transport mechanisms in *B. pendula*. The presence of acetates, formates, malonates, oxalates and citrates in leaves should be considered as a future subject of research, in order to determine, for example, the pathway of cation complexation and further migration and transport of ions to leaves. It should also be noted that the results of phosphates obtained in this study are very interesting because an easy mechanism of PO_4_^3−^ migration from soil to leaves, regardless of the area of sampling (WNP and LU), was observed. The results were confirmed by the statistical analysis as well as by bioaccumulation and translocation factors.
